# Evolutionary insights into postembryonic development of adult intestinal stem cells

**DOI:** 10.1186/2045-3701-1-37

**Published:** 2011-11-16

**Authors:** Atsuko Ishizuya-Oka, Yun-Bo Shi

**Affiliations:** 1 Department of Biology, Nippon Medical School, Kawasaki, Kanagawa 211-0063, Japan; 2 Section on Molecular Morphogenesis, Laboratory of Gene Regulation and Development, Program in Cellular Regulation and Metabolism (PCRM), Eunice Kennedy Shriver National Institute of Child Health and Human Development (NICHD), National Institutes of Health (NIH), Bethesda, Maryland, 20892, USA

**Keywords:** adult stem cell, mammalian suckling-to-weaning transition, amphibian metamorphosis, thyroid hormone

## Abstract

In the adult vertebrate intestine, multi-potent stem cells continuously generate all of the epithelial cells throughout the adulthood. While it has long been known that the frog intestine is formed via the development of adult intestinal stem cells during thyroid hormone (TH)-dependent metamorphosis, the basic structure of the adult intestine is formed by birth in mammals and it is unclear if the subsequent maturation of the intestine involves any changes in the intestinal stem cells. Two recent papers showing that B lymphocyte-induced maturation protein 1 (Blimp1) regulates postnatal epithelial stem cell reprogramming during mouse intestinal maturation support the model that adult intestinal stem cells are developed during postembryonic development in mammals, in a TH-dependent process similar to intestinal remodeling during amphibian metamorphosis. Since the formation of the adult intestine in both mammals and amphibians is closely associated with the adaptation from aquatic to terrestrial life during the peak of endogenous TH levels, the molecular mechanisms by which the adult stem cells are developed are likely evolutionally conserved.

## Introduction

The epithelium of the mammalian intestine rapidly undergoes cell-renewal originating from stem cells localized near the bottom of crypts throughout the adulthood. The descendants of the stem cells actively proliferate, gradually differentiate into absorptive cells, goblet cells, endocrine cells, and Paneth cells as they migrate along the crypt-villus axis, and finally undergo apoptosis at the tip of villi. Thus, the adult stem cells are key players in the maintenance of epithelial cell-renewal and are also targets for regeneration and/or cancer therapies. While a growing number of studies have identified many markers for the adult stem cells and key signaling pathways involved in the epithelial cell-renewal in the mammalian adult intestine [1,2], it still remains a mystery from what cells and how the adult stem cells are formed during development. Comparative studies of the expression of protein arginine methyltransferase 1 (PRMT1) have led to the model that adult intestinal stem cells are formed via conserved, thyroid hormone (TH)-dependent mechanisms in vertebrates during postembryonic development [3,4]. Two recent papers by Harper *et al*. [5] and Muncan *et al*. [6] provided convincing evidence for the formation of adult intestinal stem cells from neonatal intestinal epithelium during postembryonic development, opening the door to clarifying the molecular mechanisms underlying stem cell development during mammalian intestinal maturation.

### Mammalian intestinal maturation during suckling and weaning

During vertebrate embryogenesis, the primitive gut is generated from the definitive endoderm and the splanchnic lateral mesoderm, and then differentiates into several digestive organs including the intestine. In the mouse intestine, morphogenesis of villi starts around embryonic day 14.5 (E14.5) [2,5]. Then, the epithelial proliferation becomes restricted to the intervillus pockets, where the crypts are formed by invagination of the epithelium during the first postnatal week. Harper *et al*. [5] and Muncan *et al*. [6] recently showed that the transcriptional repressor, B lymphocyte-induced maturation protein 1 (Blimp1), is strongly expressed in the entire intestinal epithelium before birth. Then, as the plasma TH level rises, in the neonatal intestine, its expression becomes down-regulated only in the intervillus pockets where the embryonic/neonatal intestinal stem cells reside, whereas the other epithelial cells (suckling-type cells) continue to express Blimp1. The intervillus cells lacking Blimp1 expression (adult cells) form crypts and then, during suckling-to-weaning transition when the circulating TH level becomes the highest [7], the adult cells replace the suckling-type cells concomitantly with global changes in enzyme expression. Moreover, by using *VillinCre-Blimp1 *intestinal knockout mice, Blimp1 was shown to delay maturation of the intestinal epithelium. Interestingly, in contrast to down-regulation of Blimp1 expression only in the intervillus pockets and/or crypts where the stem cells are localized, the expression of candidate stem cell markers, hedgehog (hh) including Shh and Ihh [2] and a coactivator for TH receptor, protein arginine methyltransferase 1 (PRMT1) [3], is up-regulated strictly in these regions. These results imply that the adult stem cells are distinct from the embryonic/neonatal stem cells responsible for the embryonic/fetal/suckling-type epithelial cells before weaning. On the other hand, the adult stem cells lacking Blimp1 expression are derived from the immature fetal epithelial cells expressing Blimp1 [5]. Then, what are the mechanisms underlying the transformation of the embryonic/neonatal stem cells into the adult stem cells during intestinal maturation? Recent studies on amphibian metamorphosis have suggested conserved, TH-dependent mechanisms for the formation of adult intestinal stem cells in vertebrates.

### Lesson from amphibian intestinal remodeling during metamorphosis

**Figure 1:**
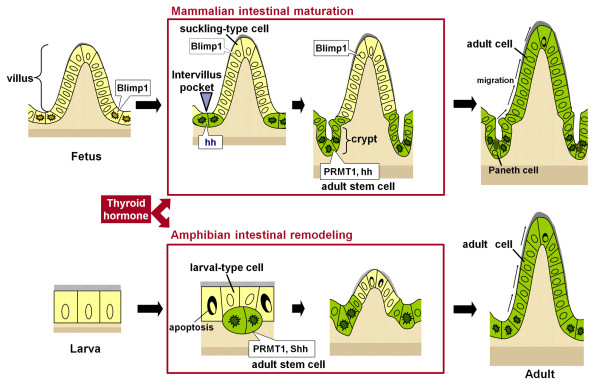
**Correlation between mouse intestinal maturation (upper panel) and *Xenopus laevis *metamorphic intestinal remodeling (lower panel)**. In both species, the adult stem cells are formed from the preexisting epithelial cells when the plasma thyroid hormone (TH) levels become high. In mouse, all epithelial cells in the fetal intestine express Blimp1 (yellow cells) and during postnatal maturation, some Blimp1 positive embryonic/fetal intestinal stem cells (yellow cells with irregular-shaped dark nuclei) in the intervillus pockets loose their Blimp1 expression and develop into adult stem cells expressing PRMT1 and hedgehog (hh) (green cells with irregular-shaped dark nuclei). In *Xenopus*, there are no identifiable stem cells for the tadpole intestinal epithelium (yellow cells) and the differentiated larval epithelial cells are mitotically active for self-renewal. During metamorphosis, most of the larval epithelial cells undergo apoptosis, whereas some undergo dedifferentiation to become the adult stem cells/progenitors that express high levels of PRMT1 and Shh (green cells with irregular-shaped dark nuclei). Subsequently, the descendants of these adult stem cells in both mouse and *Xenopus *replace the suckling-type or larval-type epithelial cells via active proliferation and differentiation to generate the adult epithelium possessing a cell-renewal system (green cells). These similarities between the both models predict evolutionary conserved TH-dependent mechanisms underlying development of the adult stem cells.

Amphibian metamorphosis reflects an evolutionary process to adapt from aquatic to terrestrial life. The *Xenopus laevis *larval-to-adult intestinal remodeling during metamorphosis has been well characterized at both the cellular and molecular levels [8]. During this period, the larval-type epithelial cells undergo apoptosis, whereas the adult stem cells expressing stem cell markers of mammalian adult intestine, e.g., the leucine-rich repeat-containing G protein-coupled receptor 5 (LGR5) and Musashi-1(Msi1), appear as islets under the control of TH and the stem cell niche. Subsequently, their descendants replace the larval-type epithelial cells by active proliferation and differentiate into the adult epithelium that acquires a cell-renewal system analogous to the mammalian one. Although it still remains unknown whether TH regulates the expression of Blimp1 in the amphibian intestine, TH directly up-regulates Shh and indirectly PRMT1 only in the adult stem cells, just like during mammalian intestinal maturation [3,8]. In addition, recombinant organ culture studies with transgenic tadpoles have shown that the adult stem cells are derived from some larval-type epithelial cells [9], similarly to the formation of the mammalian adult stem cells from the fetal epithelial cells. These similarities in the development of the adult stem cells between mammalian intestinal maturation and amphibian intestinal remodeling (Figure 1) predict that the molecular mechanisms by which the adult stem cells develop are conserved from the amphibians to mammals. Since all processes of amphibian intestinal remodeling are dependent on TH and many downstream TH response genes have been identified in the *Xenopus laevis *intestine [10], the *Xenopus *model provides an excellent opportunity to elucidate such conserved mechanisms at the molecular level. Although only fragmentary information is available about the effects of TH on the mammalian adult stem cells, accumulating evidence has shown that TH up-regulates the expression of many of the same genes such as β-catenin in the mammalian intestine as in the *Xenopus *intestine [7,10]. Thus, comparative studies of mammalian intestinal maturation and amphibian intestinal remodeling in future should pave a way for full understanding of the adult stem cells and their niche.

## Conclusion

Despite rapid progress in the stem cell biology of the mammalian adult intestine, the origin of the adult stem cells during development has been poorly understood. Recent evidence suggests that the adult stem cells are transformed from preexisting intestinal epithelial cells under the control of TH during both mammalian intestinal maturation and amphibian intestinal remodeling. Studies making use of the complementary advantages of the mammalian and frog models will shed light on the evolutionarily conserved mechanisms underlying the development of the adult stem cells, which are possibly required for the terrestrial vertebrate life in general.

## Competing interests

The authors declare that they have no competing interests.

## Authors' contributions

AI-O and YBS co-wrote and approved the manuscript.
